# Predictors of Frailty, Mild Cognitive Impairment, and Life Satisfaction Among Elderly Individuals in an Urbanized Village in New Delhi, India

**DOI:** 10.7759/cureus.114598

**Published:** 2026-08-16

**Authors:** Jeevan Jyoti Meena, Saurabh Chauhan, Abhishek Kumar, Taruna

**Affiliations:** 1 Community Medicine, Vardhman Mahavir Medical College and Safdarjung Hospital, New Delhi, IND

**Keywords:** aging, frailty, mild cognitive impairment, personal satisfaction, urban population

## Abstract

Background and aim: Aging is associated with increased vulnerability to frailty, cognitive decline, and reduced life satisfaction, particularly in rapidly urbanizing settings. This study aimed to assess the predictors of frailty, mild cognitive impairment (MCI), and life satisfaction among elderly residents in an urbanized village in New Delhi.

Materials and methods:* *We conducted a community-based cross-sectional study* *among 221 participants aged ≥60 years in South Delhi. Frailty was assessed using the Edmonton Frail Scale (EFS), cognitive function with the Montreal Cognitive Assessment (MoCA), and life satisfaction using the Satisfaction with Life Scale (SWLS). Data were analyzed using SPSS version 31.0 (Armonk, NY: IBM Corp.), and regression analysis was used to identify associations.

Results:* *The mean age was 68.4±7.2 years. Frailty was significantly associated with age ≥70 years (adjusted odds ratio {aOR}=3.72), female gender (aOR=2.27), illiteracy (aOR=2.61), chronic disease (aOR= 2.97), and MCI (aOR=6.05). MCI was linked to older age (aOR=3.04), female gender (aOR=2.48), widowhood/divorce (aOR= 10.31), illiteracy (aOR=3.98), and frailty (aOR=5.89). Lower life satisfaction was significantly associated with frailty (aOR=0.35), MCI (aOR=0.41), lower socioeconomic status (aOR=0.47), unemployment (aOR=0.48), and chronic disease (aOR=0.53).

Conclusion:* *A significant burden of frailty and cognitive impairment exists among the elderly. The strong association between physical frailty and cognitive impairment supports the conceptual framework of cognitive frailty, while the significant associations with education, socioeconomic status, and marital status emphasize the role of lifelong social determinants in shaping health trajectories in aging populations. The independent association of life satisfaction with unemployment, widowhood, and low socioeconomic status suggests the role of socioeconomic determinants in the subjective well-being of the elderly. Targeted multi-domain interventions addressing education, chronic disease management, and social support are needed to promote healthy aging in the community.

## Introduction

Aging is a natural biological process through which individuals grow older. As people age, they become increasingly vulnerable to various physical and mental health conditions. These include cardiovascular diseases, diabetes, chronic respiratory illness, musculoskeletal disorders, dementia, depression, vision loss, frailty, cognitive impairment, and other related impairments. Healthy aging involves shaping environments and opportunities that allow individuals to continue doing what matters to them throughout their lives [1].

The elderly population is increasing at an unprecedented rate worldwide, particularly in developing countries. Globally, in 2022, 1.1 billion people were aged 60 years and above, comprising 13.9% of the total global population. This number is projected to double by 2050, accounting for 22% of the total population. In India, there were 149 million persons aged 60 years and above in 2022, comprising approximately 10.5% of the total population. By 2050, the share is projected to double to 20.8%, with the absolute number rising to 347 million elderly persons. Furthermore, life expectancy at age 60 years in India is 18.3 years on average, with females living up to 19 years more, and males about 17.5 years [2].

Comprehensive geriatric assessment includes evaluating physical health (e.g., frailty), cognitive status (e.g., dementia or mild cognitive impairment), emotional well-being (e.g., depression, life satisfaction), social support, and functional capacity. This evaluation helps in creating individualized care plans and improving overall quality of life and health outcomes in the elderly population [3].

Frailty is a progressive, age-related decline in physiological systems that results in decreased reserves of intrinsic capacity, which confers extreme vulnerability to stressors and increases the risk of a range of adverse health outcomes [4]. Frailty has also been defined by Fried et al. as meeting three out of five phenotypic criteria indicating compromised energetics, including low grip strength, low energy, slowed walking speed, low physical activity, and/or unintentional weight loss [5]. The presence of one or two criteria in a prefrail stage identifies a subset with a heightened risk of advancing to frailty [5]. A meta-analysis conducted in India across healthcare settings and combined data from 6856 adults showed a prevalence of frailty of 42.3% and prefrailty of 39.8% with wide regional variation (9-76%) [6]. Various scales have been used to assess frailty, such as the Clinical Frailty Scale, Frailty Index, and Edmonton Frail Scale (EFS) [7].

Cognitive health, the ability to process, remember, and apply information, is another vital pillar of successful aging. Cognitive decline is defined as a noticeable and measurable loss or abnormality in attention functions, memory functions, or higher-level cognitive functions (including attention, language, and reasoning) [8]. Mild cognitive impairment (MCI), often a precursor to dementia, is increasingly recognized in older Indian adults, especially in communities where risk factors such as hypertension, diabetes, poor nutrition, and low education prevail [9]. The Longitudinal Aging Study in India-Diagnostic Assessment of Dementia (LASI-DAD) study reported a 17.6% prevalence of mild neurocognitive disorder (equivalent to MCI) and 7.2% for major neurocognitive disorder (dementia), indicating approximately 24 million older adults with mild impairment in India [9]. Different scales are used to measure cognitive function, such as the Mini-Mental State Examination (MMSE), Montreal Cognitive Assessment (MoCA), and Mattis Dementia Rating Scale [10]. Studies have validated the Hindi version of MoCA for use in Indian populations, demonstrating good reliability and cultural appropriateness [11].

Another essential aspect of healthy aging is life satisfaction, an emotional and psychological construct referring to an individual's cognitive evaluation of their overall life quality [12]. Life satisfaction is subject to change in terms of physical and mental health, presence of chronic diseases, friends and family relations, dependency, and events of trauma or abuse. Sociodemography, gender, education, marital status, expenditure, social support, and well-being play a pivotal role in achieving higher life satisfaction among older adults [13,14]. Older age is associated with higher life satisfaction in India, though this relationship is mediated by multiple factors, including health status and social support [15]. There are different scales to measure life satisfaction, like the Perceived Life Satisfaction Scale (PLSS), Satisfaction with Life Scale (SWLS), Patient-Reported Outcomes Measurement Information System (PROMIS) General Life Satisfaction scale, and Subjective Well-Being Inventory (SWBI) [14,15]. The SWLS has been validated in Hindi and demonstrates strong psychometric properties for use among the elderly in India [15].

Socioeconomic status, measured using tools like the Modified Kuppuswamy Scale and others, has been consistently shown to influence health outcomes in the elderly [16]. Lifestyle factors including tobacco use and alcohol consumption significantly impact aging trajectories, as do physical activity levels [17-20]. The interplay between these various determinants creates a complex web of factors that influence healthy aging. Previous research in Indian healthcare settings has documented varying prevalence rates of geriatric conditions, highlighting the need for region-specific studies [19].

Previous research has often focused on these variables independently, given the limited literature available in Indian settings. Understanding the relation between these factors is crucial for developing targeted interventions to promote healthy aging and improve the quality of life for the elderly population.

## Materials and methods

Study design and settings

This community-based cross-sectional study was conducted in an urbanized village in South Delhi, which serves as the field practice area of Vardhman Mahavir Medical College (VMMC) and Safdarjung Hospital, Delhi. The study was conducted from July 2025 to December 2025, over a period of six months.

Eligibility criteria

All individuals ≥60 years who were willing to give consent for the study were included. Participants experiencing acute illness or hospitalization at the time of data collection were excluded due to the potential impact on their frailty status, cognitive function, and life satisfaction. All elderly aged ≥60 years residing in the area for at least six months were eligible [2]. Those who were unable to understand the questionnaires in Hindi or provide informed consent due to language barriers or other communication difficulties were excluded.

Sample Size

Sample size was calculated using the below single proportion population formula.

$n = \frac{3.84&times;p&times;(1-p)}{d^2}$

Here, n=minimum sample size, p=prevalence of frailty among the elderly (42.3%), q=(1-p), and d=absolute error (7%) [6]. After substituting the values, the sample size was 191; after accounting for a 10% non-response rate, the minimum sample size was 211, and 221 study participants were included in the study.

Data collection tools

A pretested, semi-structured, and validated interview schedule was used to collect data, which consisted of the following sections: Section 1 - sociodemographic details (age, gender, caste, religion, socioeconomic status assessed using the modified Kuppuswamy scale [16]. Section 2 - lifestyle characteristics (history of substance use like tobacco use and alcohol use, history of chronic diseases, etc.) [17,18]. Section 3 - Edmonton Frailty Scale (EFS) is a validated tool that is also available in Hindi version and was used to assess frailty in older adults, for which permission was taken from the competent authority [7]. It contains nine domains, namely cognition, general health, functional independence, social support, medication use, nutrition, mood, continence, and functional performance, including simple tasks like a clock-drawing test for cognition and a basic mobility test. Each item is scored from 0 to 2, with a total score ranging from 0 to 17, classifying individuals as not frail (0-3), vulnerable (4-5), mildly frail (6-7), moderately frail (8-9), or severely frail (≥10). EFS is a comprehensive tool used to assess frailty across multiple dimensions and has been validated in diverse populations worldwide, including India, with a Cronbach's alpha of 0.71 [7,8]. Section 4 - Montreal Cognitive Assessment (MoCA) scale is a validated screening tool that evaluates key cognitive domains including visuospatial/executive functions (trail making, cube copying, clock drawing {5 points}), naming (3 animals {3 marks}), attention (6 marks), language (3 marks), abstraction (2 marks), delayed recall (5 marks), and orientation (6 marks). The test is scored out of 30, with scores ≥26 considered normal. For individuals with 12 or fewer years of education, add 1 point to adjust for educational bias. A score of <26 suggests mild cognitive impairment or early dementia and requires further evaluation. The Hindi version of the Montreal Cognitive Assessment (H-MoCA) is available and free to use [11]. MoCA has been validated in Hindi with a Cronbach's alpha of 0.64, and studies in other Indian states have demonstrated its utility in screening for cognitive impairment among the elderly [10,11]. Section 5 - satisfaction with Life Scale (SWLS) is a brief, validated tool to assess an individual's overall life satisfaction. It consists of five statements rated on a seven-point Likert scale, with total scores ranging from 5 to 35. Higher scores indicate greater life satisfaction. The respondents were asked to indicate their level of agreement with each statement using a seven-point Likert scale, where 1=strongly disagree, 2=disagree, 3=slightly disagree, 4=neither agree nor disagree, 5=slightly agree, 6=agree, 7=strongly agree. The total score is the sum of responses to all five items, with scores of 31-35 being extremely satisfied, 26-30 satisfied, 21-25 slightly satisfied, 20 neutral, 15-19 slightly dissatisfied, 10-14 dissatisfied, and 5-9 extremely dissatisfied. It has demonstrated good test-retest reliability, making it suitable for both clinical and research settings; it is also available in Hindi and is freely available [13]. SWLS has a Cronbach's alpha (α) of 0.87 and measures global cognitive judgments of one's life satisfaction across domains such as ideal life, life conditions, personal satisfaction, achievement of life goals, and willingness to relive life without changes [14]. It has been validated in Hindi with satisfactory psychometric properties [15].

Study procedure

This study was conducted in an urbanized village in South Delhi, the field practice area of VMMC and Safdarjung Hospital, Delhi, with a total population of 20,000-25,000 as per a recent survey by trained investigators. A convenience sampling technique was used to select study participants due to the absence of a comprehensive sampling frame of the elderly in the community. The first household was selected by randomly picking a direction using a random number table from the village primary health center, after which the nearest household in that direction was approached first. Subsequently, investigators moved in a fixed direction (left-to-right), visiting every consecutive household. Each household was visited sequentially, and study participants were recruited after applying inclusion and exclusion criteria until the sample size was achieved. Before the initiation of data collection, written informed consent was obtained from all participants. Face-to-face interviews were conducted, and data were collected using Google Forms (Mountain View, CA: Google LLC) with encryption enabled to protect the confidentiality of participants' responses. For anonymity, participants' names or other directly identifying information were not collected in the Google Form. They were assigned a unique identification number, and the data collected through Google Forms were stored in a secure, password-protected Google Drive (Mountain View, CA: Google LLC) account, ensuring that the responses were kept confidential (Figure 1). If the household had more than one elderly person, then only one individual was selected using the lottery method. If the house was locked or the study participant was unavailable, the next consecutive house was selected. For multistory houses, each occupied flat was considered a separate household and visited sequentially. Study participants found to be suffering from any ill health were referred to the nearby health facility for further management.

**Figure 1:**
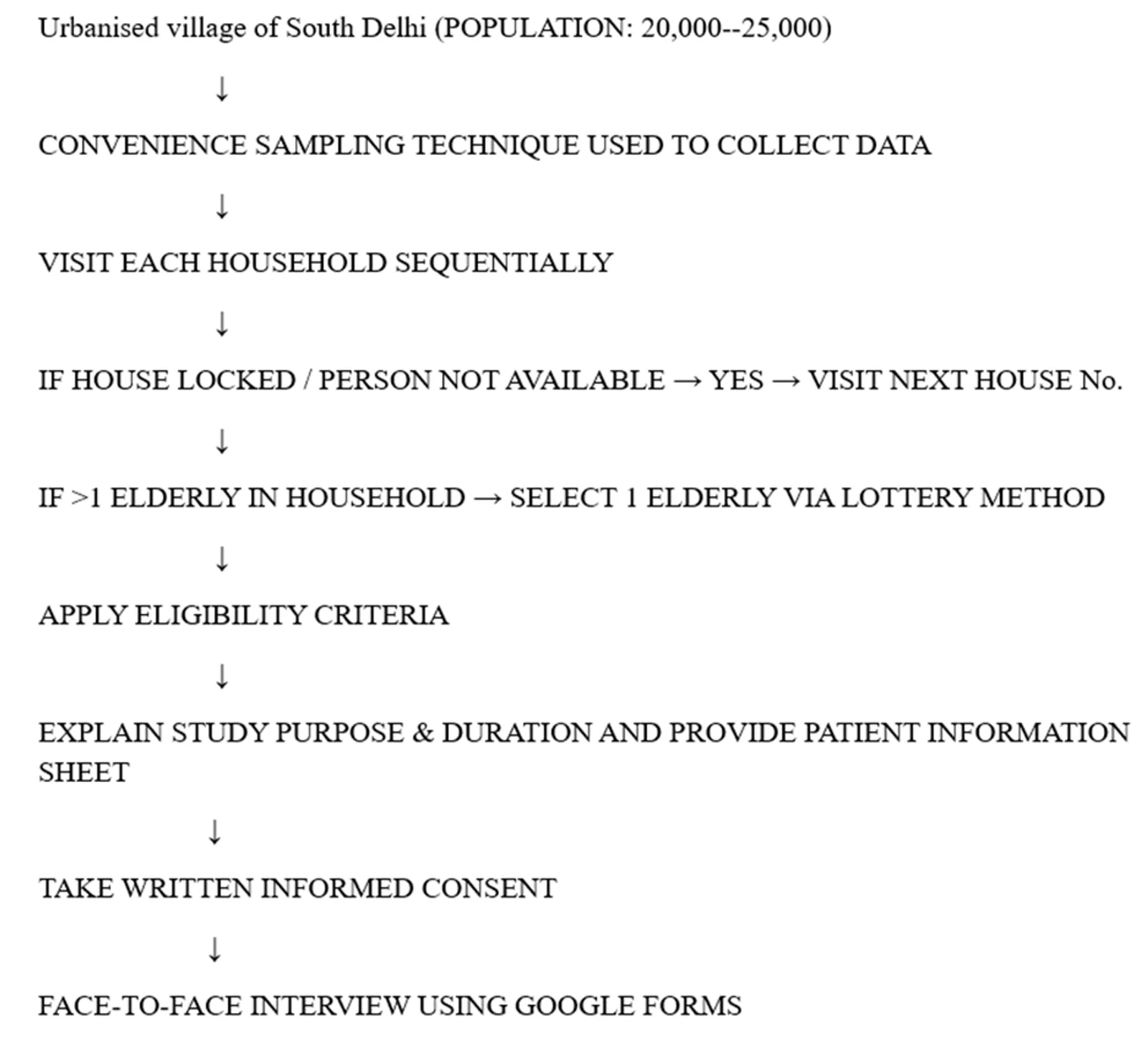
Participant flow and data collection.

Data analysis

The data were collected, compiled, and entered into Microsoft Excel (Redmond, WA: Microsoft Corp.). The data were cleaned for errors and missing values. Data analysis was carried out using licensed SPSS version 31.0 software (Armonk, NY: IBM Corp.). The results were presented in the form of tables and appropriate diagrams. Normality tests were performed, and qualitative variables were summarized as frequencies and percentages, while quantitative variables were expressed as means and standard deviations. We used multivariate regression analysis, ordinal logistic regression, and Firth's penalized logistic regression to assess associations between variables. A p<0.05 was considered statistically significant.

## Results

The mean age of the study participants was 68.4±7.2 years. The majority were aged 60-65 years (45.7%), male (61.5%), married (85.9%), and Hindu (97.3%). Unemployment was high (74.7%), and 39.8% were illiterate. The largest socioeconomic group belonged to the lower-middle class (41.3%), while 68.3% reported chronic disease and 66.5% had a history of falls (Table 1).

**Table 1:** Sociodemographic and lifestyle characteristics of study participants (n=221).

Variables	n (%)
Age categories (years)	
60-65	101 (45.7)
66-70	67 (30.3)
71-75	28 (12.8)
76-80	17 (7.6)
>80	8 (3.6)
Gender	
Male	136 (61.5)
Female	85 (38.5)
Marital status	
Married	192 (85.9)
Divorced	4 (1.8)
Widow	25 (11.3)
Religion	
Hinduism	215 (97.3)
Others (Islam, Christianity, and Sikhism)	6 (2.7)
Caste	
General	80 (36.4)
OBC	62 (27.7)
SC	53 (24.1)
ST	26 (11.8)
Occupation	
Gainfully employed	56 (25.3)
Unemployed	165 (74.7)
Education status	
Illiterate	88 (39.8)
Primary school	21 (9.5)
Middle school	40 (18.1)
High school	48 (21.7)
Intermediate/diploma	13 (5.9)
Graduate	11 (5)
Socioeconomic status (Modified Kuppuswamy Scale, 2025)	
Upper	11 (5)
Upper middle	60 (27.2)
Lower middle	91 (41.3)
Upper lower	58 (25.9)
Lower	1 (0.6)
Number of children	
<2	5 (2.3)
≥2	216 (97.7)
Any form of tobacco use	
Yes	72 (32.8)
No	149 (67.2)
Alcohol use	
Yes	39 (17.6)
No	104 (47.1)
Unwilling to tell	78 (35.3)
History of chronic disease	
Yes	151 (68.3)
No	70 (31.7)
History of falls	
Yes	147 (66.5)
No	74 (33.5)

As shown in Table 2, multivariate logistic regression identified several independent predictors of frailty. Participants aged ≥70 years had nearly three times higher odds of being frail (adjusted odds ratio {aOR}=3.72, 95% CI: 2.01-6.88, p<0.001). The prevalence of frailty was significantly higher among women as compared with men (aOR=2.27, 95% CI: 1.29-3.99, p=0.005). Widowed or divorced individuals had over four times higher odds of frailty (aOR=4.53, 95% CI: 1.96-10.49, p<0.001). Illiteracy (aOR=2.61, 95% CI:1.49-4.58, p<0.001), unemployment (aOR=1.92, 95% CI: 1.03-3.59, p=0.041), the presence of chronic disease (aOR=2.97, 95% CI: 1.59-5.56, p<0.001), and history of falls (aOR=1.83; 95% CI: 1.02-3.28) were significant drivers of frailty. Screening for MCI was the strongest correlate of frailty, increasing the odds nearly sixfold (aOR=6.05; 95% CI: 3.34-10.96; p<0.001). Here, p denotes the p-value, with values <0.05 considered statistically significant.

**Table 2:** Association of frailty with sociodemographic and lifestyle characteristics (n=221). *Multivariate regression. Ref: reference; Unadj.: unadjusted; Adj.: adjusted; SE: standard error; MCI: mild cognitive impairment

Variable (reference category)		Frail, n (%)	Not frail, n (%)	Unadjusted OR (95% CI)	β (SE)	p-Value (Unadj.)	Adjusted OR (95% CI)*	p-Value (Adj.)
Age (years)	Ref: <70	45 (26.8)	123 (73.2)	1.0	-	-	1.0	-
	≥70	38 (71.7)	15 (28.3)	3.68 (2.01-6.74)	1.30 (0.31)	<0.001	3.72 (2.01-6.88)	<0.001
Gender	Ref: male	41 (30.1)	95 (69.9)	1.0	-	-	1.0	-
	Female	42 (49.4)	43 (50.6)	2.27 (1.28-4.01)	0.82 (0.29)	0.005	2.27 (1.29-3.99)	0.005
Marital status	Ref: married	63 (32.8)	129 (67.2)	1.0	-	-	1.0	-
	Divorced/widowed	20 (69.0)	9 (31.0)	4.53 (1.95-10.55)	1.51 (0.43)	<0.001	4.53 (1.96-10.49)	<0.001
Caste	Ref: general	25 (31.3)	55 (68.8)	1.0	-	-	1.0	-
	Others (OBC/SC/ST)	58 (41.1)	83 (58.9)	0.85 (0.47-1.52)	-0.16 (0.29)	0.582	0.85 (0.47-1.53)	0.585
Occupation	Ref: employed	15 (26.8)	41 (73.2)	1.0	-	-	1.0	-
	Unemployed	68 (41.2)	97 (58.8)	1.92 (1.03-3.59)	0.65 (0.32)	0.042	1.92 (1.03-3.59)	0.041
Education	Ref: literate	38 (28.6)	95 (71.4)	1.0	-	-	1.0	-
	Illiterate	45 (51.1)	43 (48.9)	2.62 (1.50-4.59)	0.96 (0.28)	<0.001	2.61 (1.49-4.58)	<0.001
Socioeconomic status	Ref: upper/upper middle	15 (21.1)	56 (78.9)	1.0	-	-	1.0	-
	Lower middle	38 (41.8)	53 (58.2)	1.35 (0.78-2.35)	0.30 (0.28)	0.284	1.35 (0.77-2.36)	0.294
	Upper lower/lower	30 (50.8)	29 (49.2)	2.14 (1.17-3.91)	0.76 (0.30)	0.011	2.14 (1.19-3.85)	0.011
Tobacco use	Ref: no	54 (36.2)	95 (63.8)	1.0	-	-	1.0	-
	Yes	29 (40.3)	43 (59.7)	1.18 (0.67-2.09)	0.17 (0.29)	0.558	1.19 (0.67-2.11)	0.557
Alcohol use	Ref: no	38 (36.5)	66 (63.5)	1.0	-	-	1.0	-
	Yes	17 (43.6)	22 (56.4)	1.36 (0.68-2.73)	0.31 (0.35)	0.376	1.36 (0.69-2.70)	0.376
	Unwilling to tell	28 (35.9)	50 (64.1)	0.89 (0.51-1.56)	-0.12 (0.29)	0.680	0.89 (0.51-1.55)	0.678
Chronic disease	Ref: no	15 (21.4)	55 (78.6)	1.0	-	-	1.0	-
	Yes	68 (45.0)	83 (55.0)	2.98 (1.59-5.58)	1.09 (0.32)	<0.001	2.97 (1.59-5.56)	<0.001
History of falls	Ref: no	21 (28.4)	53 (71.6)	1.0	-	-	1.0	-
	Yes	62 (42.2)	85 (57.8)	1.82 (1.02-3.27)	0.60 (0.30)	0.046	1.83 (1.02-3.28)	0.043
MCI status	Ref: normal	26 (20.5)	101 (79.5)	1.0	-	-	1.0	-
	MCI	57 (60.6)	37 (39.4)	6.06 (3.35-10.98)	1.80 (0.30)	<0.001	6.05 (3.34-10.96)	<0.001

Table 3 revealed that frailty (aOR=0.35, p<0.001), MCI (aOR=0.41, p<0.001), widowhood/divorce (aOR=0.32, p<0.001), unemployment (aOR=0.48, p=0.005), lower socioeconomic status (aOR=0.47, p=0.012), chronic disease (aOR=0.53, p=0.011), and history of falls (aOR=0.55, p=0.028) emerged as significant independent factors of lower life satisfaction. Age, gender, education, and substance use were non-significant after adjustment. It was noted that unemployment and low socioeconomic status (SES) became significant only in the adjusted model, with the SES estimate even reversing direction from a non-significant positive crude effect (OR=1.41) to a significant negative adjusted effect (aOR=0.47), indicating that confounding health-related factors initially masked their true effects upon ordinal logistic regression.

**Table 3:** Association of life satisfaction with sociodemographic and lifestyle characteristics (n=221). *Ordinal logistic regression Ref: reference; Unadj.: unadjusted; Adj.: adjusted; SE: standard error; MCI: mild cognitive impairment

Variable (reference category)		Unadjusted OR (95% CI)	β (SE)	p-Value (Unadj.)	Adjusted OR (95% CI)*	p-Value (Adj.)
Age (years)	Ref: <70	1.0	-	-	1.0	-
	≥70	0.62 (0.33-1.15)	-0.478 (0.316)	0.130	0.79 (0.55-1.14)	0.206
Gender	Ref: male	1.0	-	-	1.0	-
	Female	0.83 (0.44-1.55)	-0.189 (0.320)	0.555	0.71 (0.43-1.17)	0.179
Marital status	Ref: married	1.0	-	-	1.0	-
	Divorced/widowed	0.32 (0.16-0.62)	-1.147 (0.343)	<0.001	0.32 (0.18-0.57)	<0.001
Caste	Ref: general	1.0	-	-	1.0	-
	Others (OBC/SC/ST)	0.59 (0.27-1.26)	-0.536 (0.391)	0.170	0.65 (0.39-1.08)	0.098
Occupation	Ref: employed	1.0	-	-	1.0	-
	Unemployed	0.55 (0.28-1.04)	-0.607 (0.331)	0.067	0.48 (0.29-0.80)	0.005
Education	Ref: literate	1.0	-	-	1.0	-
	Illiterate	0.61 (0.33-1.16)	-0.488 (0.323)	0.131	0.67 (0.41-1.09)	0.109
Socioeconomic status	Ref: upper/upper middle	1.0	-	-	1.0	-
	Lower middle	0.61 (0.32-1.18)	-0.491 (0.333)	0.140	0.67 (0.41-1.10)	0.112
	Upper lower/lower	1.41 (0.50-3.99)	0.344 (0.531)	0.517	0.47 (0.26-0.85)	0.012
Children	Ref: <2	1.0	-	-	1.0	-
	≥2	0.83 (0.09-7.61)	-0.186 (1.130)	0.869	0.83 (0.28-2.47)	0.735
Tobacco use	Ref: no	1.0	-	-	1.0	-
	Yes	0.92 (0.46-1.87)	-0.080 (0.359)	0.824	0.96 (0.55-1.66)	0.877
Alcohol use	Ref: no	1.0	-	-	1.0	-
	Yes	1.44 (0.64-3.20)	0.362 (0.409)	0.376	1.19 (0.63-2.24)	0.592
	Unwilling to tell	0.91 (0.53-1.56)	-0.094 (0.276)	0.733	0.91 (0.55-1.52)	0.727
Chronic disease	Ref: no	1.0	-	-	1.0	-
	Yes	0.68 (0.35-1.29)	-0.393 (0.331)	0.235	0.53 (0.33-0.86)	0.011
History of falls	Ref: no	1.0	-	-	1.0	-
	Yes	0.74 (0.37-1.49)	-0.303 (0.357)	0.396	0.55 (0.32-0.94)	0.028
Frailty	Ref: not frail	1.0	-	-	1.0	-
	Frail	0.18 (0.09-0.35)	-1.715 (0.346)	<0.001	0.35 (0.21-0.59)	<0.001
MCI status	Ref: normal	1.0	-	-	1.0	-
	MCI	0.38 (0.20-0.72)	-0.968 (0.328)	0.003	0.41 (0.24-0.69)	<0.001

Table 4 summarizes the factors associated with MCI, which were fitted using Firth's logistic regression. In adjusted analysis, age ≥70 years (aOR=3.04, 95% CI: 1.78-5.19, p<0.001), female gender (aOR=2.48, 95% CI: 1.44-4.28, p<0.001), and widowhood/divorce (aOR=10.31, 95% CI: 1.73-61.44, p<0.006) emerged as strong predictors. Illiteracy substantially increased the odds of MCI (aOR=3.98, 95% CI: 2.29-6.92, p<0.001). Lower socioeconomic status (aOR=2.48, 95% CI: 1.35-4.55, p=0.003), chronic disease (aOR=3.52, 95% CI: 1.91-6.49, p<0.001), and frailty (aOR=5.89, 95% CI: 3.25-10.65, p<0.001) were also significantly associated with MCI.

**Table 4:** Association of mild cognitive impairment (MCI) with sociodemographic and lifestyle characteristics (n=221). *Firth's logistic regression Ref: reference; Unadj.: unadjusted; Adj.: adjusted; SE: standard error

Variable (reference category)		MCI+, n (%)	Normal, n (%)	Unadjusted OR (95% CI)	β (SE)	p-Value (Unadj.)	Adjusted OR (95% CI)*	p-Value (Adj.)
Age	Ref: <70	47 (28.0)	121 (72.0)	1.0	-	-	1.0	-
	≥70	47 (88.7)	6 (11.3)	3.12 (1.75-5.56)	1.14 (0.29)	<0.001	3.04 (1.78-5.19)	<0.001
Gender	Ref: male	46 (33.8)	90 (66.2)	1.0	-	-	1.0	-
	Female	48 (56.5)	37 (43.5)	2.54 (1.48-4.36)	0.93 (0.28)	<0.001	2.48 (1.44-4.28)	<0.001
Marital status	Ref: married	66 (34.4)	126 (65.6)	1.0	-	-	1.0	-
	Divorced/widowed	28 (96.6)	1 (3.4)	53.9 (7.15-406.7)	3.99 (0.90)	<0.001	10.31 (1.73-61.44)	0.006
Caste	Ref: general	21 (26.3)	59 (73.8)	1.0	-	-	1.0	-
	Others (OBC/SC/ST)	73 (51.8)	68 (48.2)	1.53 (0.83-2.84)	0.43 (0.31)	0.165	1.52 (0.83-2.80)	0.172
Occupation	Ref: employed	16 (28.6)	40 (71.4)	1.0	-	-	1.0	-
	Unemployed	78 (47.3)	87 (52.7)	2.25 (1.21-4.19)	0.81 (0.31)	0.009	2.21 (1.19-4.11)	0.012
Education	Ref: literate	39 (29.3)	94 (70.7)	1.0	-	-	1.0	-
	Illiterate	55 (62.5)	33 (37.5)	4.07 (2.33-7.10)	1.40 (0.28)	<0.001	3.98 (2.29-6.92)	<0.001
Socioeconomic status	Ref: upper/upper middle	17 (23.9)	54 (76.1)	1.0	-	-	1.0	-
	Lower middle	42 (46.2)	49 (53.8)	1.29 (0.75-2.22)	0.25 (0.28)	0.372	1.26 (0.73-2.19)	0.412
	Upper lower/lower	35 (59.3)	24 (40.7)	2.54 (1.38-4.68)	0.93 (0.31)	0.003	2.48 (1.35-4.55)	0.003
Tobacco use	Ref: no	59 (39.6)	90 (60.4)	1.0	-	-	1.0	-
	Yes	35 (48.6)	37 (51.4)	1.44 (0.82-2.52)	0.36 (0.29)	0.214	1.42 (0.81-2.49)	0.220
Alcohol use	Ref: no	43 (41.3)	61 (58.7)	1.0	-	-	1.0	-
	Yes	19 (48.7)	20 (51.3)	1.36 (0.68-2.70)	0.31 (0.35)	0.376	1.33 (0.67-2.63)	0.417
	Unwilling to tell	32 (41.0)	46 (59.0)	0.91 (0.53-1.58)	-0.09 (0.28)	0.748	0.90 (0.52-1.56)	0.710
Chronic disease	Ref: no	16 (22.9)	54 (77.1)	1.0	-	-	1.0	-
	Yes	78 (51.7)	73 (48.3)	3.61 (1.96-6.64)	1.28 (0.31)	<0.001	3.52 (1.91-6.49)	<0.001
History of falls	Ref: no	24 (32.4)	50 (67.6)	1.0	-	-	1.0	-
	Yes	70 (47.6)	77 (52.4)	1.90 (1.08-3.36)	0.64 (0.29)	0.027	1.87 (1.06-3.29)	0.030
Frailty	Ref: not frail	37 (26.8)	101 (73.2)	1.0	-	-	1.0	-
	Frail	57 (68.7)	26 (31.3)	6.06 (3.35-10.98)	1.80 (0.30)	<0.001	5.89 (3.25-10.65)	<0.001

## Discussion

The community-based study contributes to the growing evidence on geriatric health in rapidly urbanizing, resource-limited settings in India. The high proportion of unemployment reflects the retirement status among Indian elderly, consistent with national estimates from the Longitudinal Aging Study in India (LASI) wave 1, which reported that only 24.7% of older adults were currently employed, as reported by Perkins et al. [21]. The illiteracy rate of 39.8% in our sample is comparable to the 38.2% illiteracy reported among the elderly in the Longitudinal Aging Study in India-Diagnostic Assessment of Dementia (LASI-DAD) nationally representative study, which documented that low education is a major risk factor for neurocognitive disorders in India. This finding aligns with the systematic review by Debnath et al., in which low educational status predicted frailty, showing a significantly higher prevalence of frailty among those with no formal education [6]. The predominance of lower-middle socioeconomic status (41.3%) in our sample reflects the socioeconomic profile of urbanized village populations in Delhi, which often experience transitional economies with limited access to healthcare resources, a pattern similar to findings from a study by Vadanere et al. [22]. The history of falls reported by 66.5% of participants is notably higher than the 42% prevalence reported in a study by Das, possibly reflecting the hazardous environmental conditions in urbanized villages with inadequate infrastructure and poorly maintained pathways [19].

In the present study, age was significantly associated with frailty. This finding is consistent with the meta-analysis by Debnath et al., which demonstrated that advancing age is the strongest non-modifiable risk factor for frailty, with pooled prevalence increasing from 28% in those aged 60-69 years to 52% in those aged ≥80 years across Indian studies [6]. Female gender emerged as a significant predictor, corroborating the systematic review findings that Indian women have 45% frailty prevalence compared to 35% in men. This gender disparity has been attributed to biological factors, including lower muscle mass and bone density, as well as social determinants such as differential access to nutrition and healthcare. International research from the Women's Health and Aging Studies has also consistently documented higher frailty rates among older women across diverse populations, as reported by de Jesus et al. [23]. Widowhood/divorce showed a strong association with frailty, aligning with evidence from Perkins et al., who analyzed LASI data and found that widowed individuals had significantly higher odds of poor health outcomes, with the effect being more pronounced among women due to compounded social disadvantages including economic marginalization and social exclusion [21]. Illiteracy and unemployment were significant predictors, consistent with life course social mobility research by Ko et al., which demonstrated that a consistently low socioeconomic position across the life course was associated with accelerated physical decline in old age [24]. MCI demonstrated the strongest association with frailty, supporting the concept of "cognitive frailty" as a distinct clinical entity. The observed association between frailty and mild cognitive impairment in the present study supports the evidence linking cognitive and functional decline among older adults, as done by Tsoy et al. [25].

Life satisfaction showed inverse and significant associations with frailty and MCI, which reinforces the concept of cognitive frailty aligning with LASI-based evidence studies linking physical and cognitive decline to diminished subjective well-being demonstrated by Gross et al. [9], Bramhankar et al. [13], and Das [19]. The profound negative impact of widowhood/divorce mirrors national findings attributing this to cumulative social and economic marginalization, as carried out by Paul et al. and Perkins et al. [12,21]. It was noted that unemployment and low SES became significant negative predictors only after health adjustment, with a suppressor effect because their true impact was initially masked by their strong association with poor physical and cognitive health, emphasizing cumulative socioeconomic disadvantage as shown by Bramhankar et al. and Ko et al. [13,24]. Age, gender, and education lost significance after full adjustment, suggesting their effects are largely mediated through proximate health and social determinants, as done in studies conducted by Paul et al. and Isaacson et al. [12,26]. Chronic disease and falls further reduced satisfaction, supporting evidence that physical impairments erode well-being through functional limitations and reduced social participation, as reflected by findings of Das and Yang et al. [19,27]. These findings indicate that life satisfaction in the aging population is a complex interaction of physical, cognitive, social, and economic factors.

Age emerged as a significant predictor of MCI, consistent with the LASI-DAD study by Gross et al., which reported that the prevalence of mild neurocognitive disorder increased from 12.4% in those aged 60-69 to 28.7% in those aged ≥80 years [9]. International evidence from Tsoy et al. also demonstrated that advancing age was the strongest risk factor for cognitive impairment across diverse populations [25]. The female gender showed an association with MCI, corroborating findings from the study by Dhanalakshmi et al., who reported higher cognitive impairment prevalence among elderly women, attributed to gender disparities in educational opportunities and lifelong cognitive stimulation [28]. The exceptionally strong association between widowhood/divorce and MCI requires careful interpretation due to wide confidence intervals indicating small cell sizes. However, this finding aligns with the study by Jain et al., who demonstrated that widowed individuals had significantly worse cognitive functioning compared to married counterparts, with the effect persisting longer among women [29]. It was attributed to loss of cognitive stimulation from spousal interaction, increased psychological distress, and reduced social engagement following widowhood. Perkins et al. similarly documented that widowed individuals within 0 to nine years had a significantly higher risk of depression and cognitive decline [21]. Illiteracy was a potent predictor, reinforcing the cognitive reserve hypothesis. Gupta et al. validated the Hindi version of MoCA and demonstrated that education substantially influences cognitive test performance, with each additional year of education associated with a 0.8-point increase in MoCA scores [11]. The LASI-DAD study by Gross et al. reported that illiterate individuals had 3.4-fold higher odds of neurocognitive disorders compared to those with ≥10 years of education [9]. Lower socioeconomic status was significantly associated with MCI, consistent with Ko et al., who demonstrated that consistently low socioeconomic position across the life course was associated with significantly lower cognitive function as compared to consistently high position [24]. It was noted that upward mobility was protective, suggesting that interventions improving socioeconomic conditions even in later life may confer cognitive benefits. Frailty demonstrated the strongest association with MCI, indicating bidirectional relationship. This finding is consistent with Das, who reported that cognitive frailty affected 18.5% of the population and was associated with adverse health outcomes [19]. International evidence from Del Brutto et al. demonstrated that frailty significantly predicted cognitive decline over five-year follow-up, supporting the causal nature of this relationship [30]. History of falls was significantly associated with MCI, consistent with Yang et al., who found that falls result from the complex interplay of physical and cognitive factors and can initiate a functional decline spiral [27]. These findings suggest that a history of falls may serve as an important indicator of vulnerability in the elderly and may be assessed comprehensively.

Strengths of this study include the use of validated instruments (EFS, MoCA, SWLS) with established reliability in Indian populations, a community-based design that captures real-world conditions in an urbanized village setting, and a multidimensional assessment approach that examines the interrelationships between physical, cognitive, and psychological domains of aging.

However, the cross-sectional design precludes causal inference, limiting our ability to determine the temporal direction of associations. Convenience sampling limits generalizability to other populations and may introduce selection bias, though our sample characteristics are broadly comparable to those of urban elderly in North India. Unmeasured or inadequately measured confounders such as genetic predisposition, nutritional biomarkers, baseline functional status prior to aging, polypharmacy, severity and duration of chronic illnesses, quality of social support networks, and mental health history like depression or anxiety may have influenced the observed associations, contributing to residual confounding. Additionally, the use of self-reported measures for lifestyle factors, such as tobacco use and alcohol consumption, may introduce recall bias and social desirability bias.

Implications for practice and policy

The findings of the study call for integrated, community-based geriatric care models. Interventions should focus on health education programs to improve health literacy and chronic disease management, while also targeting individuals who are illiterate or from low socioeconomic status populations. Social support initiatives, especially for isolated older adults, should aim to mitigate cognitive and emotional decline through enhanced social engagement and community connectivity. Regular screening for frailty and cognitive impairment in primary care settings using validated tools can enable early identification and intervention. Chronic diseases should be managed in an integrated manner, with particular attention to their impact on both physical and cognitive function.

## Conclusions

This study highlights a significant burden of frailty and cognitive impairment among the elderly. The associations between frailty and cognitive impairment suggest that these conditions often coexist in the elderly and require comprehensive assessment, which further supports the conceptual framework of cognitive frailty, while the significant associations with education, socioeconomic status, and marital status emphasize the role of lifelong social determinants in shaping health trajectories in aging populations. The independent associations of unemployment, widowhood, and low socioeconomic status with lower life satisfaction even after accounting for health status highlight the irreplaceable role of socioeconomic determinants in subjective well-being among the elderly. Future longitudinal research and targeted multidomain interventions addressing education, chronic disease management, and social support, alongside economic empowerment and social security initiatives, are needed to promote healthy aging and enhance life satisfaction in similar settings.
